# Amelioration of human lupus-like phenotypes in MRL/*lpr* mice by overexpression of interleukin 27 receptor α (WSX-1)

**DOI:** 10.1136/ard.2007.077537

**Published:** 2007-12-18

**Authors:** N Sugiyama, H Nakashima, T Yoshimura, A Sadanaga, S Shimizu, K Masutani, T Igawa, M Akahoshi, K Miyake, A Takeda, A Yoshimura, S Hamano, H Yoshida

**Affiliations:** 1 Department of Medicine and Biosystemic Science, Graduate School of Medical Sciences, Kyushu University, Fukuoka, Japan; 2 Division of Nephrology and Rheumatology, Department of Internal Medicine, Fukuoka University, School of Medicine, Fukuoka, Japan; 3 Department of Ophthalmology, Graduate School of Medical Sciences, Kyushu University, Fukuoka, Japan; 4 Division of Molecular and Cellular Immunology, Medical Institute of Bioregulation, Kyushu University, Fukuoka, Japan; 5 Department of Medicine and Clinical Science, Graduate School of Medical Sciences, Kyushu University, Fukuoka, Japan; 6 Department of Parasitology, Graduate School of Medical Sciences, Kyushu University, Fukuoka, Japan; 7 Department of Biomolecular Sciences, Faculty of Medicine, Saga University, Saga, Japan

## Abstract

**Objective:**

In the present work, we investigate the role of interleukin (IL)27/IL27 receptor α (Rα) (WSX-1) in the development of autoimmune disorders in the MRL/*lpr* mouse, which is considered as an experimental model of systemic lupus erythaematosus (SLE) in humans.

**Methods:**

We generated two strains of WSX-1 transgenic mice in the MRL/*lpr* background with different expression levels of WSX-1, and investigated the effect of WSX-1 overexpression on survival, glomerulonephritis and immunological properties.

**Results:**

In comparison with wild type (WT) MRL/*lpr* and transgenic (Tg) low (TgL) mice, Tg high (TgH) mice exhibited a prolonged lifespan and no apparent development of autoimmune nephritis. Production of anti-dsDNA antibody and total IgG and IgG2a were significantly lower in TgH mice than those of TgL and WT mice. The expressed amounts of interferon (IFN)γ and IL4 mRNA by CD4^+^ T cells from Tg mice decreased in a dose-dependent fashion. CD4^+^ splenic lymphocytes in TgH mice were more subject to the IL27-mediated suppression of cytokine production. In vitro stimulation of CD4^+^ T cells by IL27 resulted in over phosphorylation of STAT3 in TgH cells than in WT cells.

**Conclusion:**

WSX-1 overexpression in the MRL/*lpr* background rendered the autoimmune prone mice protected from the development of autoimmune diseases. Our results suggest that IL27 signalling may be a therapeutic target against autoimmune diseases, including human SLE.

Interleukin 27 is a member of the IL6/IL12 family and is composed of a p28 subunit and Epstein-Barr virus-induced gene 3, polypeptides structurally related to p35 and p40 of IL12, respectively.^1^ IL27 is produced by activated antigen-presenting cells and induces proliferation of and T bet expression in naïve CD4^+^ T cells.^1^ ^2^ WSX-1, which was cloned as a homologue of gp130 of the IL6 receptor,^3^ constitutes a functional signal-transducting receptor for IL27 with gp130.^4^ WSX-1 is highly expressed in CD4^+^ T cells as well as in natural killer (NK)/natural killer T (NKT) cells and macrophages.^3^ ^5^ ^6^ Analysis of mice deficient for WSX-1 infected with *Leishmania major* revealed the critical role of WSX-1 in the initial mounting of proper Th1 responses.^6^ In infection with *Trichuris muris*, a nematode whose clearance depends on Th2 responses, WSX-1-deficient mice showed impaired Th1 responses with augmented Th2 responses resulting in more efficient expulsion of the worms than that in wild type (WT) mice, confirming its role for Th1 development.^7^ ^8^

Recent lines of evidence, however, have shown a distinct role for WSX-1 and its ligand, IL27, as an attenuator of inflammatory responses. In *Toxoplasma gondii* or *Trypanosoma cruzi* infection, CD4^+^ T cells as well as NKT cells and macrophages in WSX-1-deficient mice overproduced several inflammatory cytokines, resulting in devastating inflammation in the liver and other organs.^9^ ^10^ The suppressive role of WSX-1 was also observed in various experimental settings such as concanavalin A (Con A)-induced hepatitis, *Mycobacterium tuberculosis* infection, an allergic asthma model and experimental autoimmune encephalomyelitis.^11^^–^15 These data clearly demonstrated that IL27/WSX-1 plays an inhibitory role by regulating cell activation and cytokine production.^16^

Systemic lupus erythaematosus (SLE) is a multi-system disease that is caused by tissue damage resulting from autoantibody and complement-fixing immune complex deposition. Lupus nephritis manifests considerable heterogeneity in phenotype and histology. In particular, diffuse proliferative glomerulonephritis (DPGN) and membranous glomerulonephritis (MGN) represent two histological forms that are polar opposites.^17^ ^18^ The pathogenesis of DPGN is associated with predominance of Th1 cytokines,^19^ while that of MGN with predominantly Th2 cytokine response.^20^ MRL/*lpr* mice develop a systemic autoimmune disease, which is reminiscent of SLE in humans. In MRL/*lpr* mice, Fas-mediated apoptosis of activated lymphocytes was severely impaired, and T cell-dependent production of autoantibodies results in immune complex-mediated glomerulonephritis and vasculitis.^21^ ^22^ Kidney disease in MRL/*lpr* mouse is a particularly suitable model of DPGN. Intriguingly, disruption of the WSX-1 gene changed the pathophysiology of glomerulonephritis developing in MRL/*lpr* (WT) mice. WSX-1^–/–^ MRL/*lpr* mice developed a disease resembling human MGN with augmented Th2 responses, confirming that the Th1/Th2 cytokine balance is a key to the pathogenesis of differential types of glomerulonephritis.^23^ In this study, we generated lines of WSX-1 transgenic MRL/*lpr* mice to further investigate roles of IL27/WSX-1 in the development of autoimmune disorders in MRL/*lpr* mice.

## METHODS

### Generation of WSX-1 transgenic MRL/*lpr* mice

WSX-1 transgenic mice in the MRL/*lpr* background were produced by crossing WSX-1 transgenic BALB/c mice^24^ into the MRL/*lpr* background more than six times (continual backcrossing: 98.44% in MRL/*lpr* background). Genotyping for *lpr* alleles was performed by PCR as described previously.^23^ We generated two strains of *WSX-1* transgenic mice in the MRL/*lpr* background (transgenic high (TgH) and low (TgL)) depending on different expression levels of WSX-1. Female mice from the same litters were used in the present study. Mice were maintained in the Laboratory of Animal Experiments of Kyushu University. All experiments were approved by the Institutional Animal Research Committee of Kyushu University and conformed to the animal care guidelines of the American Physiologic Society.

### Western blotting

We evaluated the production of WSX-1 protein in the transgenic mice using anti-T cell lymphocyte cytokine receptor (TCCR) (WSX-1) antibody (Abcam, Cambridge, Massachusetts, USA), anti-β-actin antibody (Sigma, St Louis, Missouri, USA), and anti-mouse IgG-horseradish peroxidase (HRP) antibodies (Amersham Biosciences, Piscataway, New Jersey, USA). They were visualised with an electrochemical luminescence (ECL) detection system (Amersham Biosciences).

### Laboratory assessments

For serum chemistry, total protein, blood urea nitrogen (BUN) and creatinine (Cr)^8^ levels were assessed in the sera from 10 mice in each group at 24 weeks. Urinary protein:urinary Cr ratios were also determined. Anti-nuclear antibodies (ANA) were detected by indirect immunofluorescence using HEp-2 substrate slides (Orgentec, Mainz, Germany) with fluorescein isothiocyanate-conjugated AffiniPure donkey anti-mouse IgG (Jackson ImmunoResearch, West Grove, Pennsylvania, USA).^25^ ^26^ Serum anti-double-stranded DNA (anti-dsDNA) antibodies (Abs) were analysed by ELISA (Shibayagi, Gunma, Japan). For serum Ig, determination ELISA was performed using the following antibodies: rat anti-mouse IgG1 (Zymed Laboratories, San Francisco, California, USA), rat anti-mouse IgG1-HRP (BioSource International, Camarillo, California, USA), goat anti-mouse IgG2a (Bethyl Laboratories, Montgomery, Alabama, USA), rabbit anti-mouse IgG2a-HRP (Cappel Lab, Durham, North Carolina, USA) and goat anti-mouse IgE Ab (Bethyl Laboratories).

### Histopathological and immunohistopathological studies of kidneys

The severity of glomerulonephritis was evaluated as described previously.^23^ For immunohistochemical staining, kidneys were snap frozen in optimal cutting temperature compound (Sakura, Osaka, Japan). To detect immune complex (IC) deposits, cryostat sections (2 µm) were fixed in chilled acetone and stained with fluorescein isothiocyanate (FITC)-conjugated goat polyclonal anti-mouse IgG Abs (Organon Teknika, Scarborough, Maine, USA), a FITC-conjugated goat anti-mouse IgG1 Ab and a FITC-conjugated goat anti-mouse IgG2a Ab (Southern Biotechnology Associates, Birmingham, Alabama, USA). For negative controls, sections were treated with normal goat IgG (Santa Cruz Biotechnology, Santa Cruz, California, USA). The fluorescence strength was analysed using scion image (Scion Cooperation, Frederick, Maryland, USA).^27^

### Real-time quantitative PCR and TaqMan primers and probes

Expression levels of interferon (IFN)γ and IL4 in CD4^+^ T cells were determined using TaqMan PCR and an ABI prism 7700 sequence detection system (Applied Biosystems Japan, Tokyo, Japan). The relative expression of each mRNA was determined and normalised to the expression of the internal housekeeping gene glyceraldehyde 3-phosphate dehydrogenase (GAPDH). Primer and probe sequences are described previously.^23^

### Activation of CD4^+^ T cells

CD4^+^ T cells were purified from splenic extracts using magnetic beads (Miltenyi Biotec GmbH, Bergisch Gladbach, Germany). Purified CD4^+^ T cells were activated with plate-bound anti-CD3 Ab (1 μg/ml) plus soluble anti-CD28 Ab (1 μg/ml) (BD Biosciences, San Jose, California, USA) for 2 days, transferred to a new plate without antibodies and additionally cultured for 5 days as a total of 7 days either in the presence or absence of IL27. Culture supernatants containing recombinant murine IL27 were prepared as described previously.^16^ The cells were then washed and restimulated either with anti-CD3 Ab plus anti-CD28 Ab for cytokine production or with IL27 for signal transducer and activator of transcription (STAT) activation. Anti-STAT1, anti-phosphotyrosine (pY)-STAT1 and anti-pY-STAT3 Abs were purchased from New England BioLabs (Beverly, Massachusetts, USA). Anti-STAT3 Ab was purchased from Santa Cruz Biotechnology.

### Measurement of cytokines

Cytokines in culture supernatants of CD4+ T cells were analysed using a micro bead-based ELISA system (Multiplex Antibody Bead Kits, Biosource) according to the manufacturer’s directions with Luminex 100 (Luminex, Austin, Texas, USA). Cytokines in the sera were measured by ELISA kits (Genzyme, R&D Systems, Abingdon, UK and eBioscience, Los Angeles, California, USA) for detection of IFNγ, IL4, IL17A and IL2.

### Statistical analyses

For survival of mice, Kaplan–Meier analysis was carried out using the Statview software package (SAS Institute Japan, Tokyo, Japan). Other quantitative data were expressed as the mean (SD). The Mann–Whitney U rank sum test was performed to analyse the difference between two groups, while for individual comparisons among the three groups the Kruskal–Wallis test, followed by the Scheffe test was performed. All tests were two-tailed. A p value of less than 0.05 was considered statistically significant.

## RESULTS

### Prolonged survival in TgH mice, not in TgL mice

**Figure 1:**
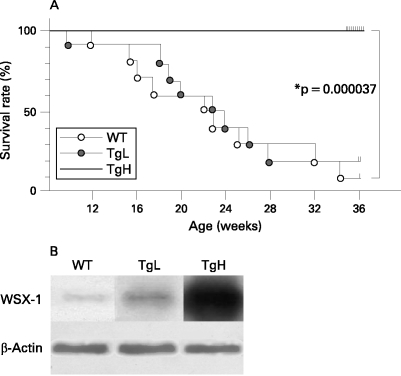
Prolonged survival of MRL/*lpr* mice with high expression of WSX-1. A. The cumulative survivals of wild type (WT), transgenic low (TgL) and high (TgH) mice were monitored weekly (n = 20 per group). *p<0.05 by the Kaplan–Meier method. There was no difference in the survival rate between WT and TgL mice. B. Western blot analysis of the transgenic expression of WSX-1 in CD4^+^ T cells from WT mice and two Tg-positive MRL/*lpr* mouse lines, TgL and TgH. The lysates of purified splenic CD4^+^ T cells from 16-week-old WT, TgL and TgH mice were separated by sodium dodecyl sulfate polyacrylamide gel electrophoresis (SDS-PAGE) under reducing condition and transferred to membranes. Expression levels for β-actin protein was visualised for each sample as a loading control. The transgene products demonstrate robust, moderate and faint expression of WSX-1 in TgH, TgL and WT mice T cells, respectively.

The MRL/*lpr* mice develop a rapid and fluminant autoimmune nephritis with 50% mortality at 6 months of age.^28^ In this study MRL/*lpr* mice (WT mice) died after birth at a rate described above. Although TgL mice died at the similar rate as WT mice, TgH mice showed significantly extended survival rates (0% dead at 24 weeks after birth; p<0.001 over WT mice) (fig 1A).

### Improvement of clinical features and parameters in TgH mice

Given the striking improvement of survival in TgH mice, clinical features and serum chemistry parameters of 24-week-old mice were examined. TgH mice showed significantly lower BUN, compared with WT and TgL mice (table 1). Additionally, the urinary protein:creatinine ratio was also lower in TgH mice than in TgL or WT mice. These data indicated amelioration of kidney function in TgH mice. The parameters of 36-week-old TgH mice were comparable with those of 24-week-old TgH mice (data not shown). Of note, splenomegaly and lymphadenopathy were also significantly reduced in TgH mice.

**Table 1:** Clinical manifestations and serum chemistry in female wild type (WT), transgenic low (TgL) and high (TgH) mice

	MRL/*lpr* 24W		
	WT	TgL	TgH
Body weight, g	49.0 (6.10)	43.1 (4.40)	40.4 (3.00)
Spleen weight, g	1.31 (0.56)	0.90 (0.21)	0.33 (0.07)*
Total lymph node weight, g	6.14 (1.71)	3.43 (3.31)	1.51 (0.53)*
Urinary protein:creatinine ratio	17.4 (5.80)	15.98 (10.6)	5.24 (2.22)*
Serum protein, g/dl	6.83 (0.67)	6.93 (0.66)	7.68 (0.77)
Blood urea nitrogen, mg/dl	57.2 (24.9)	57.9 (19.2)	28.4 (6.00)*
Serum creatinine, mg/dl	0.27 (0.12)	0.20 (0.09)	0.36 (0.14)

Values are mean (SD).

*p<0.05 vs WT

### Amelioration of glomerulonephritis in TgH mice

**Figure 2:**
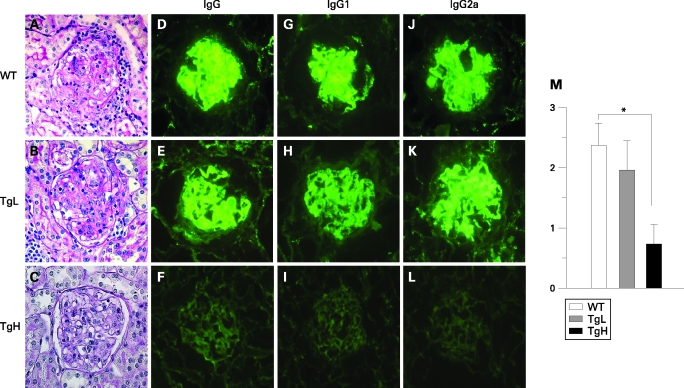
Amelioration of glomerulonephritis and decreased Ig deposition in transgenic high (TgH) mice. The kidneys were fixed in 10% formalin for 24 h at 4°C. Paraffin sections (4 µm) were stained either with H&E, periodic acid Schiff stain, or periodic acid-methenamine silver (PAM). Microscopic examination of the kidney glomerulus of a 24-week-old wild type (WT) mouse (A), transgenic low (TgL) mouse (B) and TgH mouse (C) (periodic acid Schiff staining; original magnification, ×400). IgG (D, E and F), IgG1 (G, H and I) and IgG2a (J, K and L) deposits in the glomeruli of a 24-week-old WT, TgL and TgH mice were visualised using immunofluorescent anti-Ig staining (×400) and quantitative analysed by image software. WT: IgG (83.8 (7.25)), IgG1 (68.4 (6.4)) and IgG2a (76.8 (4.9)), TgL: IgG (81.0 (8.48)), IgG1 (71.4 (7.3)) and IgG2a (73.0 (4.6)), TgH:IgG (25.2 (9.65)), IgG1 (22.8 (6.45)), IgG2a (19.6 (3.2)). These representative data obtained from 30 glomerular cross sections per kidney (six mice per group) with similar staining patterns. M. The severity of glomerulonephritis was evaluated by the score of glomerular proliferative activity. The scores of glomerular proliferative activity of 24-week-old WT, TgL and TgH mice were 2.38 (0.356), 1.92 (0.564) and 0.74 (0.378), respectively.

Since kidney dysfunction is the primary cause of death in MRL/*lpr* mice, histopathological examination of kidneys from the three groups of 24-week-old mice was performed. Typical histological features of DPGN were observed in WT and also in TgL mice, including inflammatory cell infiltration, glomerular sclerosis, mesangial proliferation and crescent formation (fig 2A,B). By striking contrast, TgH mice showed drastic attenuation of inflammatory and proliferative changes (fig 2C). The score of glomerular proliferative activity of TgH was significantly decreased compared to that of WT (fig 2M. p = 0.0074 TgH vs WT).

Deposition of immunoglobulin is one of the hallmarks of glomerulonephritis.^15^ We then performed immunofluorescent staining of the kidneys to detect Ig in glomeruli in the three groups of mice. An intense IgG deposition was detected in mesangial lesions and along the capillary walls of glomeruli in WT and TgL mice. The isotypes of the deposited Ig were mainly IgG2a and IgG1 in part (fig 2D,E,G,H,J,K). By contrast, IgG deposition was hardly observed in TgH mice, and the deposition of IgG1 and IgG2a was remarkably decreased (fig 2F,I,L), and the fluorescent strength score was significantly decreased (p = 0.0035 TgH vs WT).

### Decreased production of ANA, anti-dsDNA antibodies and immunoglobulins

**Figure 3:**
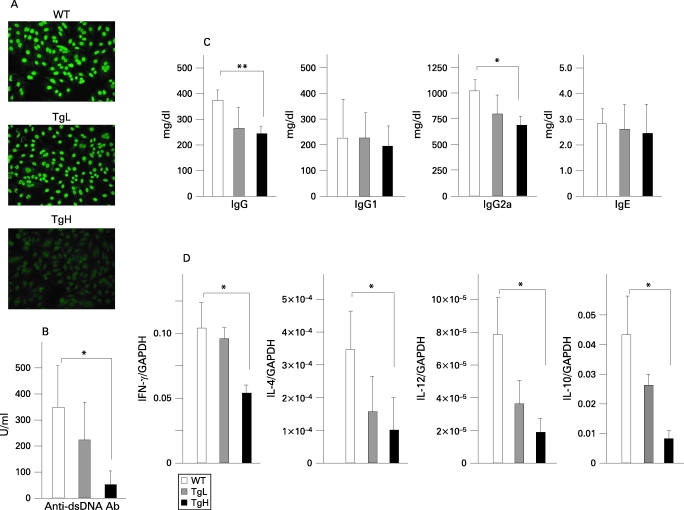
Decreased autoreactive immune responses in transgenic high (TgH) mice. A. Sera collected from 16-week-old wild type (WT), transgenic low (TgL) and TgH mice were examined for anti-nuclear antibodies (ANA) as described in Materials and methods. The same sera in (A) were measured for anti-dsDNA antibodies (B) or for Ig levels (C). D. The expression levels of interferon (IFN)γ and interleukin (IL)4 mRNA in CD4^+^ T cells and those of IL12 and IL10 mRNA in B220^+^CD3^+ ^cell-depleted splenocytes were examined by quantitative PCR. Data shown are mean (SD) of 10 mice per group. *p<0.05 by unpaired Student t test.

To further examine the immunological changes in TgH mice, the level and the nature of serum Ig and autoantibodies were evaluated. Positive staining for ANA was detected in 16-week-old WT TgL mouse sera at a 1:200 dilution, as determined by indirect immunofluorescence (n = 10 per group), whereas sera from 16-week-old TgH mice were negative for ANA (n = 8) (fig 3A). The sera from 24- and 36-week-old TgH mice were also negative (data not shown). Production of anti-dsDNA Abs was significantly lower in TgH mice than those of WT and TgL mice (fig 3B). While there were no significant differences in the levels of IgG1 and IgE, the levels of total IgG and IgG2a were significantly lower in TgH mice than in other groups (fig 3C).

### Reduced cytokine production by CD4^+^ T cells from TgH mice

Expression of IFNγ and IL4 in splenic CD4^+^ T cells decreased in a manner dependent on the expression levels of WSX-1 (fig 3D). Similarly, expression of IL12b and IL10 in spleen cells depleted of B220^+^CD3^+ ^cell was also decreased in *WSX-1* transgenic mice.

### Decrease in CD3^+^B220^+^CD4^–^CD8^–^ T cell, and increase in CD4^+^ and CD8^+^ T cell in the WSX-1 TgH MRL/*lpr* mouse

**Figure 4:**
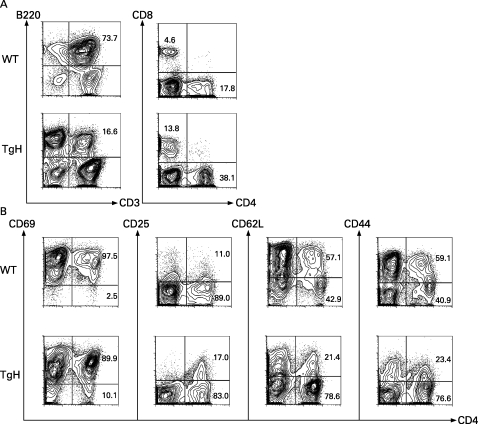
Decrease in CD3^+^B220^+^CD4^–^CD8^–^ T cell and upregulated expression of activation markers on transgenic high (TgH) CD4^+^ T cells. A. Spleens were removed from 16-week-old wild type (WT) and TgH mice, and single cell suspensions were stained for the expression of surface markers, followed by flow cytometry. Numbers are the percentage of CD3^+^B220^+^CD4^–^CD8^–^ T cells, CD8^+^ T cells and CD4^+^ T cells against total cell populations. B. Surface expression levels of CD69, CD25, CD62L and CD44 were analysed by flow cytometry. Numbers are the percentage of the total CD4^+^ T cells.

Given the significant improvement of splenomegaly and lymphadenopathy in TgH mice (table 1), cellular composition in the spleen was analysed. The percentage of CD3^+^B220^+^CD4^–^CD8^–^ T cells in TgH mouse was greatly diminished over WT mice and in TgL mice (fig 4A, and data not shown). Concomitantly, percentages of CD4^+^ and CD8^+^ T cells increased compared with WT mice.

### Expression of activation markers in CD4^+^ T cells in WSX-1 TgH MRL/*lpr* mice

To clarify the activation status of CD4^+^ T cells in the mice, expression of several cell surface activation markers was evaluated (fig 4B). In WT and TgH mice, most of the CD4^+^ T cells expressed CD69 on their cell surface, due presumably to in their possible autoreactivity. More than 10% of CD4^+^ T cells were positive for CD25 expression in WT and TgH mice. While some 40% of CD4^+^ T cells did not express CD62L in WT mice, approximately 80% of CD4^+^ T cells did not express CD62L. Interestingly, however, less CD4^+^ T cells expressed CD44 in TgH mice than in WT mice. These data demonstrated that while most CD4^+^ T cells were activated by CD69 expression in WT and TgH mice, more cells in TgH mice showed the activated phenotype by CD62L expression. However, because much fewer cells expressed CD44 in TgH than in WT mice, the CD4^+^ T cells in TgH mice appeared to be in an activation status different from that in WT mice. Although the percentage of CD25^+^ cells in the CD4^+^ T cell population was higher in TgH mice than in WT mice, there was no significant difference in the expression levels of FoxP3 in CD4^+^ T cells between these mice (data not shown).

### CD4^+^ T cells from WSX-1 TgH mice were more subject to the IL27-mediated suppression of cytokine production

To examine the effect of IL27 on lymphocyte activity in the mice, cytokine production by in vitro activated CD4^+^ T cells either in the presence or absence of IL27 was examined (fig 4A). CD4^+^ T cells from TgH mice produced more IFNγ and less IL4 than those from WT mice. Nonetheless, CD4^+^ T cells from TgH mice were more sensitive to IL27-mediated suppression of cytokine production. Although WT and TgH CD4^+^ T cells were subject to IL27-mediated suppression of IFNγ and IL4 production, the suppressive effect was prominent in TgH cells over WT cells. Production of IFNγ and IL4 was strikingly suppressed to barely detectable levels in TgH CD4^+^ T cells. IL17 production by TgH CD4^+^ T cells was lower than WT cells even without IL27 addition, and was suppressed to a barely detectable level in TgH cells. Interestingly, IL2 production was not affected by WSX-1 overexpression and CD4^+ ^cells from WT and TgH mice were similarly subject to IL27-mediated suppression.

**Figure 5:**
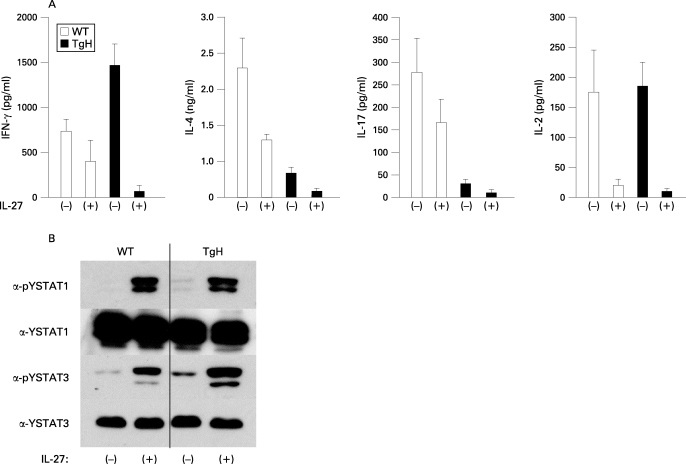
IL27 suppression of cytokine production by activated CD4^+^ T cells. A. CD4^+^ T cells from wildtype (WT) and transgenic high (TgH) mice were stimulated with plate-bound anti-CD3 antibody plus soluble anti-CD28 antibody (1 μg/ml) in the absence or presence of interleukin (IL)27 for 24 h. Culture supernatants were measured for the production of respective cytokines. Data shown are mean (SD) of 10 mice per group. *p<0.05 by unpaired Student t test. B. CD4^+^ T cells from WT and TgH mice were stimulated as in Materials and methods. Phosphorylation of signal transducer and activator of transcription (STAT)1 or STAT3 in whole cell lysates in WT and TgH mice was analysed with anti-phosphotyrosine (α-pY) -STAT1 or anti-pY-STAT3 antibodies, respectively. The filter was stripped and re-probed with anti-STAT1 or anti-STAT3 antibodies to ensure the same amounts of samples were loaded. Experiments were repeated three times with similar results.

Downstream of the IL27R (WSX-1 plus gp130), STAT1 and STAT3 are activated.^16^ ^24^ When CD4^+^ T cells were isolated and immediately examined for STAT1/3 phosphorylation, phosphorylation of STAT1 and STAT3 was apparent in TgH CD4^+^ T cells as compared with WT cells (fig 5B). When these cells were stimulated with IL27 for 1 h, further phosphorylation of STAT1 and STAT3 was observed in WT and Tg cells. While the levels of STAT1 phosphorylation were comparable between WT and Tg cells, STAT3 phosphorylation was higher in Tg cells than in WT cells.

## DISCUSSION

In this study we generated lines of Tg mice that overexpressed WSX-1 in T cells to examine the impact of WSX-1 overexpression on pathophysiology and autoimmune status of MRL/*lpr* mice. We demonstrated that overexpression of WSX-1 suppressed the development of autoimmune nephritis in WT mice, and ANA, the values of anti-dsDNA Ab, serum Ig and expression of various cytokines, significantly decreased in of the Tg mice. While CD4^+^ T cells in TgH mice were in a distinct activation status from those in WT mice, these cells were more subject to the effects of IL27 in vitro. These results strongly suggested that increased expression of WSX-1 suppressed the autoimmune reaction and the subsequent glomerulonephritis in WT mice.

We originally demonstrated the pivotal role of WSX-1 in the initial mounting of Th1 differentiation via T bet induction,^2^ and in mice deficient in the WSX-1 gene, proper Th1 differentiation was impaired with Th2 skewing during protozoan infection.^6^ In line with these findings, we revealed that disruption of the *WSX-1* gene drastically changed the histological features of glomerulonephritis developing in MRL/*lpr* mice from DPGN to MGN accompanied by impaired IFNγ production with predominance Th2-dependent IgG1 deposition and increased levels of IgG1 and IgE in the sera.^23^ T cells in *WSX-1*^–/–^ MRL/*lpr* mice displayed spontaneous skewing of autoimmune responses toward Th2 type. Thus, our previous reports suggested that immune status, or more specifically, the balance between Th1 and Th2 responses, is a key determinant for the pathogenesis of the glomerulonephritis. Counterintutively, transgenic overexpression of *WSX-1* gene resulted in amelioration of glomerulonephritis in MRL/*lpr* mice. It would be reasonable to assume this was the result of the suppressive effects of IL27 since the cytokine expression by CD4^+^ T cells as well as autoimmune reaction was suppressed in TgH mice.

CD4^+^ T cells from TgH mice activated in vitro produced more IFNγ and less IL4 than those from WT mice and showed Th1 phenotyping, which was in line with the Th1-promoting function of IL27. However, IL27 addition strikingly suppressed production of IFNγ and IL4 to barely detectable levels in Tg CD4^+^ T cells (fig 5A). These results confirmed the suppressive effect of IL27 on cytokine production and also revealed that the suppressive effect was much higher for CD4^+^ T cells of TgH than for those of WT mice. Such suppression of cellular response was largely consistent with the diminished pathophysiology of glomerulonephritis in TgH mice. We have recently reported that IL27 exerts its suppressive effects preferentially on activated CD4^+^ T cells, and that STAT3 activation in response to IL27 stimulation of activated T cells is, at least partially, responsible for the IL27-mediated suppression of cytokine production.^16^ This is quite consistent with our present finding that TgH mice CD4^+^ T cells were more sensitive to IL27 stimulation by STAT3 activation (fig 5B). Ohwaki *et al* reported the involvement of STAT1 in IL27-mediated suppression of IL2 production by naïve T cells,^29^ although Villarino *et al* reported that IL27-mediated suppression in activated T cells is independent of STAT1.^30^ The discrepancy may be ascribed to the activation status of the cells.

In summary, we demonstrated that WSX-1 overexpression in the MRL/*lpr* background rendered the autoimmune prone mice protected from the development of autoimmune disease. Further elucidation of the molecular mechanisms underlying the IL27-mediated cytokine suppression and detailed dissection of the situations where IL27 differentially exerts its two roles will no doubt help development of new therapies against various diseases by suppressing excess of cell responses.
